# Supplementary material for: Dual-Enzyme Cascade Composed of Chitosan Coated FeS2 Nanozyme and Glucose Oxidase for Sensitive Glucose Detection
Source: Molecules. 2023 Jan 31;28(3):1357. doi: 10.3390/molecules28031357 (PMC9919173; doi:10.3390/molecules28031357)
Supplement: Supplementary file 1 [file molecules-28-01357-s001.zip › molecules-2155627-supplementary.pdf]

Table S1. Immobilized enzyme efficiency

Materials	Amout of Gox (mg)	Immobilized enzyme Efficiency (%)	Amount of immobilized enzyme (mg)
FeS_2	0.1	15.2	0.0312
FeS_2	0.3	16.6	0.0738
FeS_2	0.5	20.3	0.1015
FeS_2	0.75	12.2	0.0915
FeS_2	1	9.8	0.098
FeS_2/CS	0.1	70.2	0.0702
FeS_2/CS	0.3	75.4	0.2262
FeS_2/CS	0.5	80.3	0.4015
FeS_2/CS	0.75	51.5	0.35625
FeS_2/CS	1	30.3	0.303

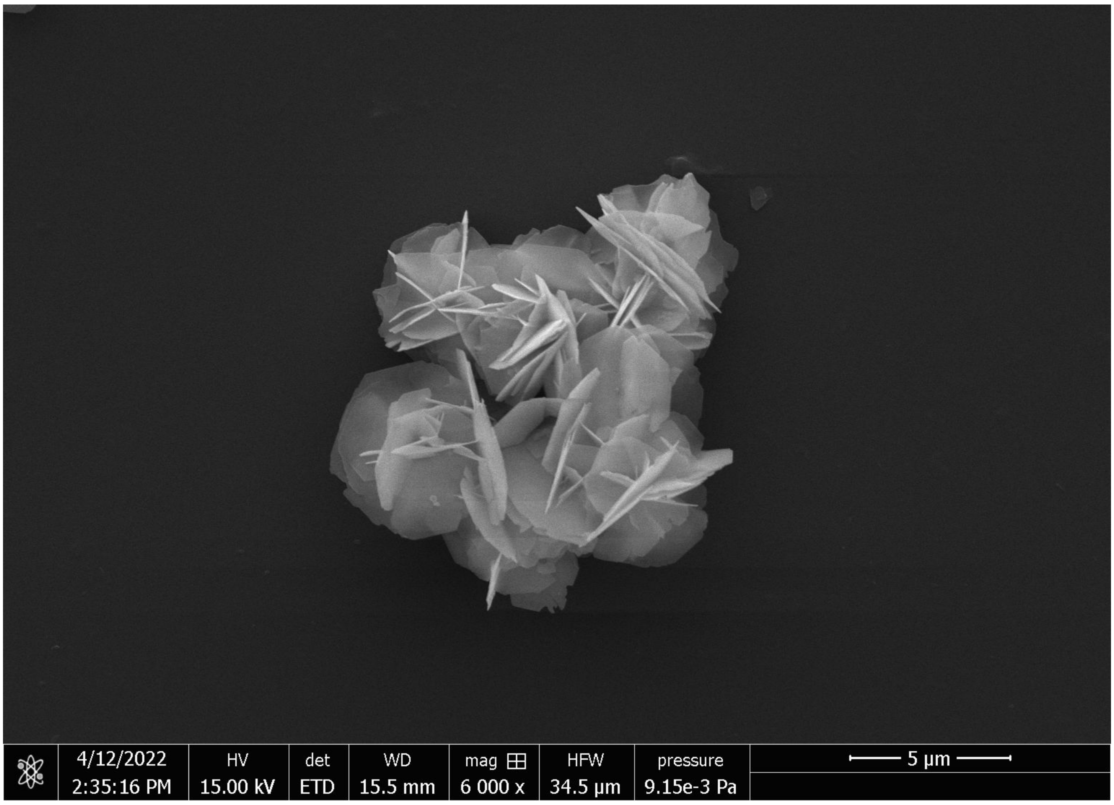

Figure S1. Morphology of FeS_2

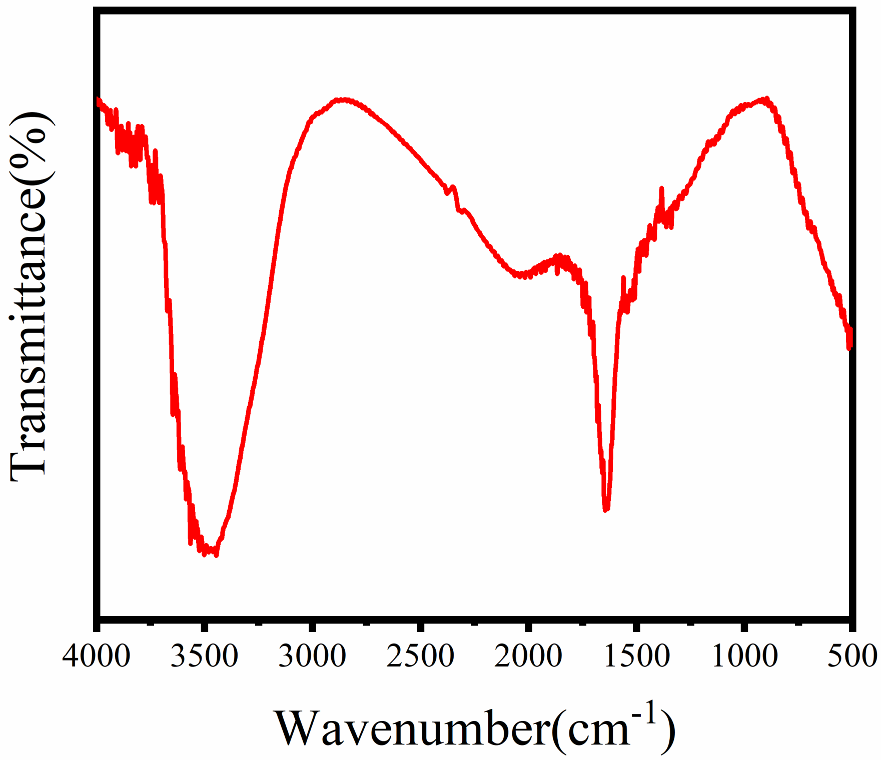

Figure S2. FTIR absorption spectrum of Gox.

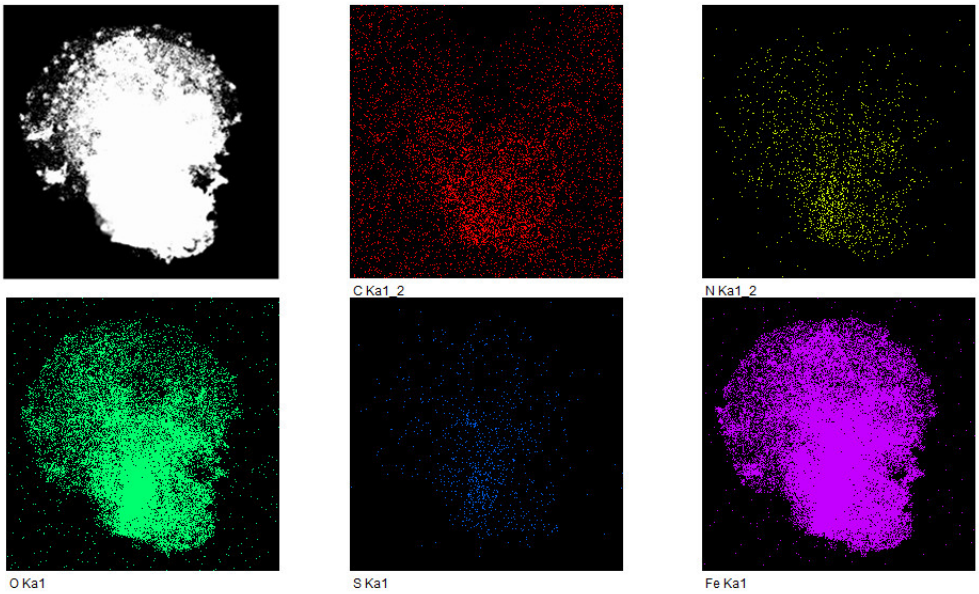

Figure S3. Elemental distribution of FeS_2/CS@Gox.

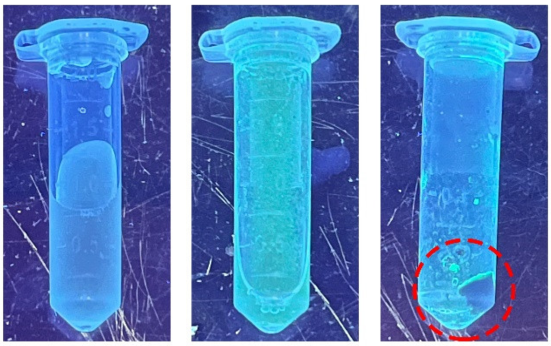

Figure S4. The Color of FeS_2/CS@Gox under UV light.

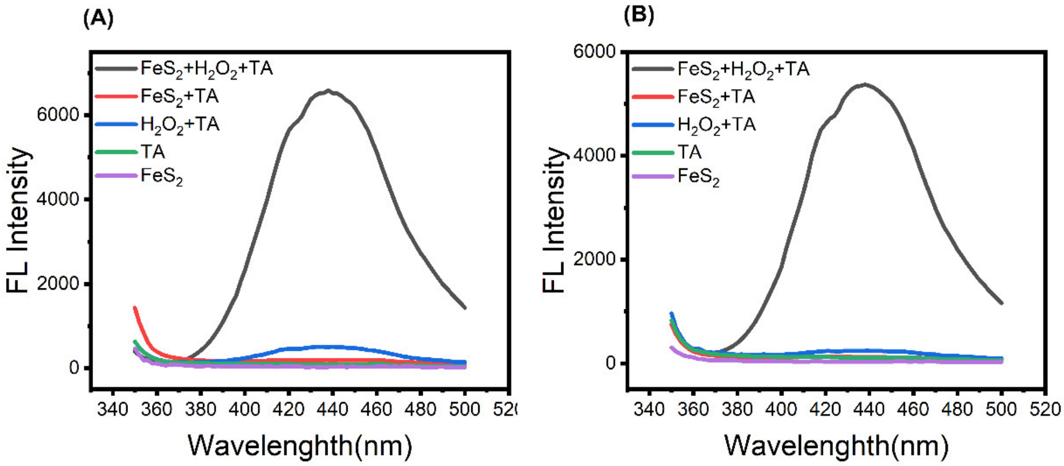

Figure S5. Hydroxyl radicals produced by FeS_2.

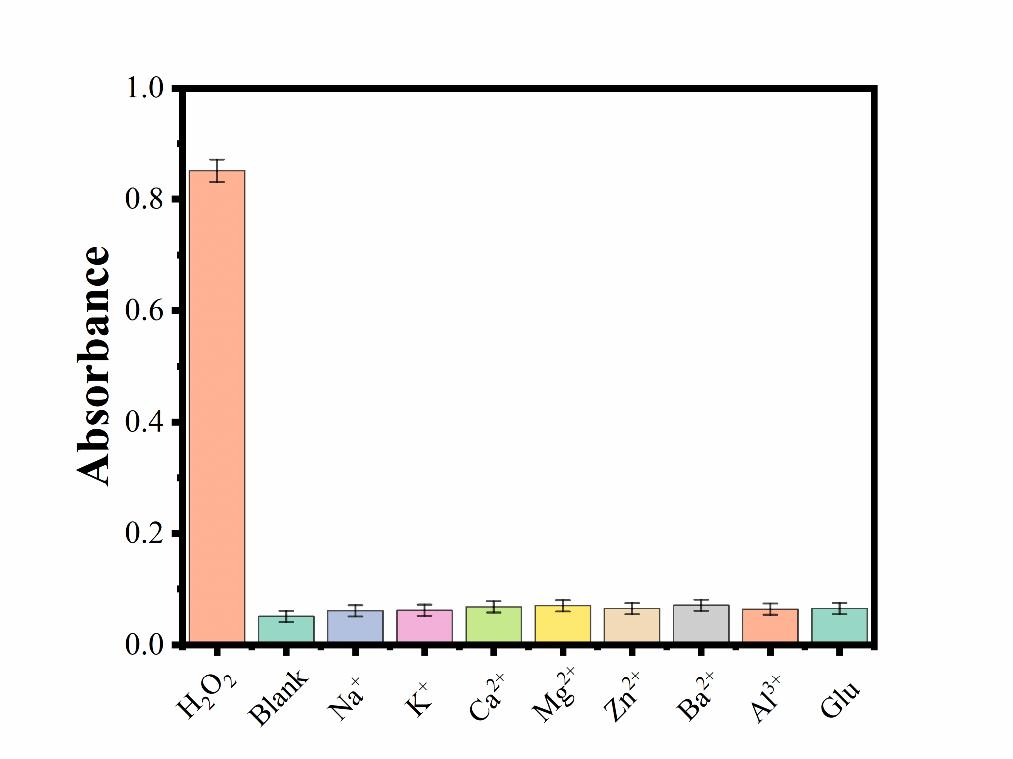

Figure S6. Selectivity of FeS_2 to H_2O_2.

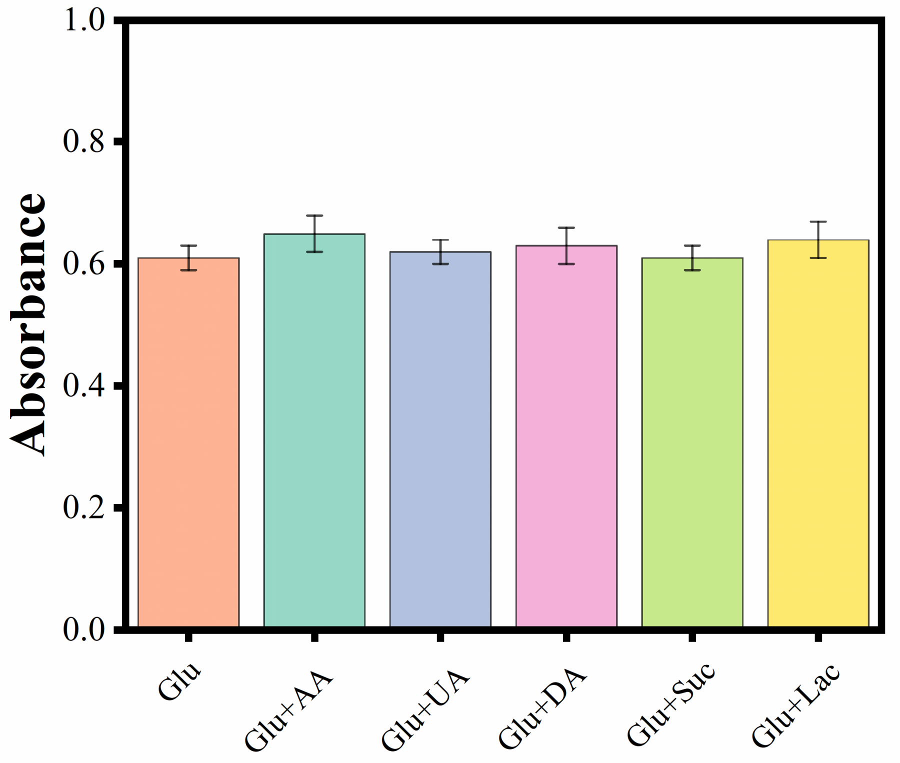

Figure S7. Selectivity of FeS_2/CS@Gox to glucose.

AA: ascorbic acid; UA: uric acid; DA: dopamine; Suc: sucrose; Lac: lactic acid。
